# Association between self‐perception of general health and sun‐related behaviours

**DOI:** 10.1002/ski2.304

**Published:** 2023-10-13

**Authors:** Cherna Cherfrere, Alexander Wu, Cristina Thomas

**Affiliations:** ^1^ Department of Internal Medicine University of Texas Southwestern Medical Center Dallas Texas USA; ^2^ University of Texas Southwestern Medical School University of Texas Southwestern Medical Center Dallas Texas USA; ^3^ Department of Dermatology University of Texas Southwestern Medical Center Dallas Texas USA

Dear Editor,

Ultraviolet (UV) radiation is a major risk factor for the development of melanoma and non‐melanoma skin cancer.^1^ Although preventative measures, including wearing sunscreen and protective clothing, are known to reduce skin cancer risk^2^ and have increasingly been used over the past decade, general public use of these sun‐protective behaviours still remains low.^3^ Previous research has shown that a patient's risk perception (i.e. risk of being diagnosed with skin cancer) can affect patient adherence to sun‐protective behaviours, and that self‐risk perception varies significantly based on demographic variables. For example, black, older, and less educated populations perceive a lower risk of developing skin cancer.^4^, ^5^ Patients' self‐perception of their general health has also been shown to affect preventative health‐related behaviours.^6^ However, little is known about the effect of patients' self‐perception of general health on sun‐related behaviours that can increase skin cancer risk. Using the Health Information National Trends Survey (HINTS) 5 Cycle 3, we examined whether self‐perceived general health is associated with sun‐related behaviours.

HINTS 5 cycle 3 is a national cross‐sectional survey of US adults aged 18 years and older administered from January to May 2019. Data were collected from 5438 correspondents, with participants defining perception of their general health by selecting “excellent”, “very good”, “good”, “fair”, and “poor”. To evaluate sun‐protective behaviours, the following questions were analysed: “On warm sunny days, how often do you spend time in the sun in order to get a tan” (Tanning vs. No Tanning), “To what extent do you enjoy spending time in the sun” (Don't Enjoy vs. Enjoy), “Number of times sunburned in the past year” (No Sunburn vs. Sunburned in the past year), and “The most recent time you got sunburned, were you doing any of the following things to protect yourself from the sun: 1) Wearing sunscreen with SPF of at least 15, 2) Wearing protective clothing such as long pants or a shirt with sleeves that cover your shoulders, or 3) Staying in the shade or under an umbrella”. Frequencies and Chi‐square tests were used to determine categorical differences between those with positive perceptions (defined as excellent, very good, good responses) and negative perceptions (defined as fair and poor) of general health. Dichotomisation of ordinal exposure and outcome variables was performed in order to calculate odds ratios. Weighted univariate and multivariate logistic regression odds ratios and 95% confidence intervals (95% CI) were used to determine associations between sun‐related behaviours and perception of general health. Responses were weighted per HINTS statistical recommendation. Statistical analysis was performed using SAS v9.4 (SAS Institute, Inc.). This study was not subject to Institutional Review Board review.

Of the 5438 responses, completed data for demographic variables were collected from 4661 respondents. Of these 4661 respondents, 2671 were female (57.3%), and the largest age group of respondents was aged 60 years or older (46.7%). Most participants had a self‐reported ethnicity of white, non‐Hispanic (62.2%), had an income of less than $50,000 (44.4%), and were college graduates (47.0%). Age (*p* = 0.0047) and race/ethnicity (*p* = 0.0004) were found to be significantly associated with different perceptions of one's general health, as younger respondents aged 18–39 and non‐Hispanic white or black respondents were more likely to report better general health. Additionally, respondents with incomes over $20,000 (*p* < 0.0001) and graduates of high school or higher (*p* < 0.0001) were also more likely to perceive their health positively. Gender was not significantly associated with general health perceptions (*p* = 0.5965) (Table S1). After performing multivariable analysis and adjusting for demographic characteristics and other sun‐related behaviours, having a positive perception of one's general health resulted in higher odds of a history of tanning (aOR: 1.70, CI: 1.15–2.50) and being sunburned (aOR: 1.79, CI: 1.15–2.78) (Table 1). A positive perception of one's general health was also associated with sunscreen use (uOR: 2.16, CI: 1.22–3.80) and enjoying spending time in the sun (uOR: 2.29, CI: 1.49–3.53) in the univariate analysis but not in the multivariable analysis.

**TABLE 1:** Weighted univariate and multivariate logistic regression models for positive self‐perceptions of general health and sun‐related behaviours.

	Univariate analysis^a^				Multivariate analysis^a^			
Characteristics	Good or better^b^		Fair or worse^b^		Good or better^b^		Fair or worse^b^	
	uOR (95% CI)	*p* Value	uOR (95% CI)	*p* Value	aOR (95% CI)	*p* Value	aOR (95% CI)	*p* Value
Tanning^c^	1.85 (1.42–2.41)	<0.0001	1.00 (ref)	‐	1.70 (1.15–2.50)	<0.01	1.00 (ref)	‐
Enjoy spending time in sun^d^	2.29 (1.49–3.53)	<0.01	1.00 (ref)	‐	1.66 (0.96–2.86)	0.07	1.00 (ref)	‐
Sunburned in the past year^e^	2.45 (1.73–3.47)	<0.01	1.00 (ref)	‐	1.79 (1.15–2.78)	0.01	1.00 (ref)	‐
Use of sun‐ protective behaviours during the last sunburn^f^	1.37 (0.68–2.76)	0.38	1.00 (ref)	‐	1.16 (0.50–2.67)	0.73	1.00 (ref)	‐
Sunscreen use	2.16 (1.22–3.80)	<0.01	1.00 (ref)	‐	1.84 (0.84–4.04)	0.12	1.00 (ref)	‐
Wearing protective clothing in the sun	0.89 (0.27–2.96)	0.85	1.00 (ref)	‐	0.76 (0.15–3.96)	0.74	1.00 (ref)	‐
Staying in the shade	1.22 (0.66–2.26)	0.53	1.00 (ref)	‐	1.16 (0.59–2.29)	0.66	1.00 (ref)	‐

^a^ Both Univariate logistic regression analysis and Multivariable logistic regression analysis were weighted using HINTS statistical recommendations, and the multivariate logistic regression analysis was adjusted for all variables listed in Table 1 (Age, Sex, Race/Ethnicity, Education, and Income).

^b^ Positive perception was defined as answering “Excellent”, “Very good”, or “Good” to the question “In general, would you say your health is…”, whereas negative perception was defined as answer “Fair” or “Poor” to the same question.

^c^ Defined as answering “Rarely”, “Sometimes”, or “Often” to the question “On warm sunny days, how often do you spend time in the sun in order to get a tan?”

^d^ Defined as answering “A little”, “Some”, or “A lot” to the question “To what extent do you enjoy spending time in the sun?”

^e^ Defined as answering “1 or more” to the question “During the past 12 months, how many times have you had a sunburn (even a small part of your skin turns red or hurts for 12 h or more) from too much sun exposure?”

^f^ Defined as answering “Wearing sunscreen with SPF of at least 15”, “Wearing protective clothing such as long pants or a shirt with sleeves that cover your shoulders”, or “Staying in the shade or under an umbrella” to the question “The most recent time you got sunburned, were you doing any of the following things to protect yourself from the sun?”

Many factors play a role in adherence to sun‐protective behaviours including age, location of residence, knowledge, attitudes, and perceived cancer risk.^7^ We sought to evaluate whether an association between self‐perception of general health and sun‐related behaviours exists. We found that having a positive perception of one's health was associated with higher odds of a history of tanning and sunburns. The reasons for this are likely multifactorial. Individuals with a positive perception of their general health may be more active and more likely to spend time outdoors, predisposing them to sunburns.^3^ Self‐perception of health is also an important predictor of mortality, and those with a positive self‐perception of their health may be more willing to practice risky behaviours given the lower risk of overall mortality.^8^, ^9^ Even with riskier sun‐related behaviours and higher risk of skin cancer, these individuals' overall well‐being (including wellness maintenance, physical activity, healthy diet) may outweigh the potential risk of these behaviours impacting their overall health.^6^, ^9^ These findings correlate with previous studies demonstrating multiple health benefits associated with a moderate amount of sun exposure, including a decreased risk of all‐cause mortality, malignancies, diabetes, obesity, psoriasis, and cardiovascular disease.^10^ Interestingly, a positive self‐perception of health was also associated with sunscreen use and enjoying spending time in the sun, but only in the univariate analysis, suggesting that these factors may correlate to specific demographic characteristics.

Limitations of the study include the cross‐sectional nature of the survey, which prevented the study of changes in these behaviours over time, and reliance on self‐reported responses. The majority of respondents were white, non‐Hispanic and college graduates. This population may have better overall health outcomes despite risky sun‐related practices and potentially higher skin cancer risk, and thus, our results may not be generalisable to minority populations. Additionally, detailed information on the participants' views about skin health and skin cancer risks was not provided in the survey.

As efforts expand to promote sun protective behaviours and increase patient knowledge about UV exposure and skin cancer, it is important to understand how patients' perceptions about their own health affect their behaviours. Based on our results, a nuanced discussion regarding sun exposure should be held with all patients but may be particularly important in those with a positive perception of their health. The benefits of sun exposure are well‐known including a decreased risk of all‐cause mortality, decreased risk of cognitive decline, increased vitamin D production, and increased serotonergic activity.^1^, ^10^ These benefits must be balanced by the potential risk of skin cancer related to risky sun‐related behaviours. Patient counselling regarding healthy sun‐related practices should be personalised based on skin type, geographic residence, skin cancer risk, and comorbidities.

## CONFLICT OF INTEREST STATEMENT

None to declare.

## FUNDING INFORMATION

This research received no specific grant from any funding agency in the public, commercial, or not‐for‐profit sectors.

## AUTHOR CONTRIBUTIONS

**Cherna Cherfrere**: Conceptualisation (lead); Data curation (equal); Formal analysis (equal); Funding acquisition (equal); Investigation (equal); Methodology (lead); Project administration (equal); Resources (equal); Software (equal); Supervision (equal); Validation (equal); Visualisation (equal); Writing – original draft (equal); Writing – review & editing (equal). **Alexander Wu**: Conceptualisation (equal); Data curation (equal); Formal analysis (equal); Funding acquisition (equal); Investigation (equal); Methodology (equal); Project administration (equal); Resources (equal); Software (equal); Supervision (equal); Validation (equal); Visualisation (equal); Writing – original draft (equal); Writing – review & editing (equal). **Cristina Thomas**: Conceptualisation (lead); Data curation (equal); Formal analysis (equal); Funding acquisition (equal); Investigation (equal); Methodology (equal); Project administration (lead); Resources (lead); Software (equal); Supervision (lead); Validation (equal); Visualisation (equal); Writing – original draft (lead); Writing – review & editing (lead).

## ETHICS STATEMENT

Not applicable.

## Supporting information

Table S1

## Data Availability

The data that support the findings of this study are openly available at https://hints.cancer.gov/data/download‐data.aspx.
