# Supplementary material for: Association between self‐perception of general health and sun‐related behaviours
Source: Skin Health Dis. 2023 Oct 13;4(6):e304. doi: 10.1002/ski2.304 (PMC11608880; doi:10.1002/ski2.304)
Supplement: Supplementary file 1 — Table S1 [file SKI2-4-e304-s001.docx]

Table S1. Demographic characteristics by self-perception of general health

| **Characteristics** | **All** | **General Health Rating % (95% CI)** | | **P Value*** |
| --- | --- | --- | --- | --- |
|  |  | **Good or better** | **Fair or worse** |  |
| **Age group (years)** |  |  |  | 0.0047 |
| 18-39 | 31.3 (29.6-33.0) | 88.9 (85.2-92.6) | 11.1 (7.4-14.8) |  |
| 40-59 | 40.6 (38.5-42.7) | 83.7 (80.7-86.6) | 16.3 (13.4-19.3) |  |
| over 60 | 28.1 (26.5-29.7) | 81.6 (79.5-83.7) | 18.4 (16.3-20.5) |  |
| **Sex** |  |  |  | 0.5965 |
| Male | 48.9 (45.6-52.2) | 84.4 (81.8-87.0) | 15.6 (13.0-18.2) |  |
| Female | 51.1 (47.8-54.4) | 85.3 (83.4-87.2) | 14.7 (12.8-16.6) |  |
| **Race/Ethnicity** |  |  |  | 0.0004 |
| Non-Hispanic White | 66.8 (64.4-69.2) | 84.8 (80.0-89.6) | 15.2 (10.4-20.0) |  |
| Non-Hispanic Black | 11.9 (10.3-13.5 | 86.8 (85.0-88.6) | 13.2 (11.4-15.0) |  |
| Hispanic/Latino | 12.4 (10.7-14.1) | 76.3 (71.5-81.2) | 23.7 (92.2-94.9) |  |
| Other† | 8.8 (7.2-10.4) | 80.7 (75.5-85.9) | 19.3 (14.1-24.5) |  |
| **Education** |  |  |  | <.0001 |
| Less than High School | 6.9 (5.8-7.0) | 68.5 (61.3-75.8) | 31.5 (24.2-38.7) |  |
| High School Graduate | 23.4 (21.4-25.4) | 81.5 (77.2-85.7) | 18.5 (14.3-22.8) |  |
| Some college | 40.0 (37.4-42.6) | 83.0 (80.0-86.0) | 17.0 (14.0-20.0) |  |
| College Graduate | 29.6 (27.7-31.5) | 93.6 (92.2-94.9) | 6.4 (5.1-7.8) |  |
| **Income** |  |  |  | <.0001 |
| Less than 20,000 | 18.4 (16.2-20.6) | 70.7 (66.0-75.4) | 29.3 (24.6-34.0) |  |
| 20,000 to 34, 999 | 11.1 (9.6-12.5) | 79.8 (73.1-86.4) | 20.2 (13.6-26.9) |  |
| 35,000 to 49,999 | 13.5 (11.8-15.2) | 80.8 (75.9-85.7) | 19.2 (14.3-24.1) |  |
| 50,000 to 99,999 | 30.4 (28.1-32.7) | 89.3 (86.7-91.8) | 10.7 (8.2-13.3) |  |
| 100,000 or more | 26.5 (24.4-28.6) | 94.3 (92.0-96.7) | 5.7 (3.3-8.0) |  |

**Table legend**

*P values were determined using the Wald Chi-Square test

† Race/Ethnicity of “Other” includes American Indian or Alaska Native, Asian Indian, Chinese, Filipino, Japanese, Korean, Vietnamese, Other Asian, Native Hawaiian, Guamanian or Chamorro, and Samoan or Other Pacific Islander.
